# Identification of Genes Required for Long-Term Survival of *Legionella pneumophila* in Water

**DOI:** 10.1128/msphere.00454-22

**Published:** 2023-03-29

**Authors:** Philipp Aurass, Seongok Kim, Victor Pinedo, Felipe Cava, Ralph R. Isberg

**Affiliations:** a Department of Enteropathogenic Bacteria and Legionella, Robert Koch Institute, Wernigerode, Germany; b Department of Molecular Biology and Microbiology, Tufts University School of Medicine, Boston, Massachusetts, USA; c Laboratory for Molecular Infection Medicine Sweden, Department of Molecular Biology, Umeå Centre for Microbial Research, Umeå University, Umeå, Sweden; University of Wisconsin—Madison

**Keywords:** CRISPRi, *Legionella*, Tn-seq, persistence, starvation, virulence, water

## Abstract

Long-term survival of Legionella pneumophila in aquatic environments is thought to be important for facilitating epidemic outbreaks. Eliminating bacterial colonization in plumbing systems is the primary strategy that depletes this reservoir and prevents disease. To uncover L. pneumophila determinants facilitating survival in water, a Tn-seq strategy was used to identify survival-defective mutants during 50-day starvation in tap water at 42°C. The mutants with the most drastic survival defects carried insertions in electron transport chain genes, indicating that membrane energy charge and/or ATP synthesis requires the generation of a proton gradient by the respiratory chain to maintain survival in the presence of water stress. In addition, periplasmically localized proteins that are known (EnhC) or hypothesized (*lpg1697*) to stabilize the cell wall against turnover were essential for water survival. To test that the identified mutations disrupted water survival, candidate genes were knocked down by CRISPRi. The vast majority of knockdown strains with verified transcript depletion showed remarkably low viability after 50-day incubations. To demonstrate that maintenance of cell wall integrity was an important survival determinant, a deletion mutation in *lpg1697*, in a gene encoding a predicted l,d-transpeptidase domain, was analyzed. The loss of this gene resulted in increased osmolar sensitivity and carbenicillin hypersensitivity relative to the wild type, as predicted for loss of an l,d-transpeptidase. These results indicate that the L. pneumophila envelope has been evolutionarily selected to allow survival under conditions in which the bacteria are subjected to long-term exposure to starvation and low osmolar conditions.

**IMPORTANCE** Water is the primary vector for transmission of L. pneumophila to humans, and the pathogen is adapted to persist in this environment for extended periods of time. Preventing survival of L. pneumophila in water is therefore critical for prevention of Legionnaires’ disease. We analyzed dense transposon mutation pools for strains with severe survival defects during a 50-day water incubation at 42°C. By tracking the associated transposon insertion sites in the genome, we defined a distinct essential gene set for water survival and demonstrate that a predicted peptidoglycan cross-linking enzyme, *lpg1697*, and components of the electron transport chain are required to ensure survival of the pathogen. Our results indicate that select characteristics of the cell wall and components of the respiratory chain of L. pneumophila are primary evolutionary targets being shaped to promote its survival in water.

## INTRODUCTION

Legionella pneumophila is a waterborne pathogen that is the causative agent of Legionnaires’ disease, which results in pneumonia associated with high levels of lethality (1). The bacterium and related species are widespread in natural and artificial water systems, and it persists planktonically, colonizes biofilms, and infects permissive protozoa (2, 3). The colonization of municipal water systems is of great significance (4), as it is the leading cause of drinking water-related Legionnaires’ disease outbreaks in the United States (5). This facilitates infection via the main route of transmission to humans, which is inhalation of contaminated aerosols shed from poorly managed water systems. After contamination, the eradication of *L. pneumophila* from water supplies has proven challenging, so much of public health containment has been devoted to engineering water systems that prevent colonization of the pathogen. In the past 2 decades, there has been consistent increase in the number of diagnosed Legionnaires’ disease cases (6). The estimated direct health care cost of Legionnaires’ disease due to hospitalization and emergency room visits in the United States was estimated more than USD $400 million in 2014 (1).

As a waterborne pathogen, *L. pneumophila* is equipped to survive, persist, and—when associated with biofilms—grow in drinking water (4). It has the documented ability to persist in water for months or even years without losing its ability to regrow and to infect host cells (7–10). Since Legionnaires’ disease is highly seasonal in nature, the bacterium may persist for very long periods of time in water supplies prior to an epidemic outbreak of disease. The expanding burden of waterborne legionellosis illustrates the need to better understand how *Legionellae* survive in water and in protozoa in order to identify points of vulnerability and to design remediation strategies.

Previous work highlighted the importance of the stringent response and the alternative sigma factor RpoS for starvation survival in water. Key factors orchestrating its immediate adaptation to nutrient limitation include the LetA/S-rsmYZ-CsrA regulatory cascade (11–16). The CsrR protein promotes long-term survival by an as-yet-unknown mechanism (17). Other water stress survival-promoting factors include a putative metal transporter LasM and a putative transcriptional regulator Lpg2524 (18, 19). Transcriptomic or proteomic screens identified *L. pneumophila* genes upregulated under prolonged exposure to water (“water stress”) or proteins particularly abundant under these conditions (20, 21). These studies were designed to positively select for the (over)expressed subgenome under water stress conditions. Weakly transcribed or repressed genes with importance during persistence or for regrowth might escape these screening approaches.

Most of the effort in understanding the physiology of *L. pneumophila* has been devoted to identifying and determining the mechanism of action of proteins necessary for intracellular growth of the bacterium within environmental amoebae and target macrophages, which form the replicative niche during pneumonic disease in humans (22, 23). In contrast, there has been little progress in identifying proteins necessary for survival in tap water, partly because systematic analysis requires extensive incubation under starvation conditions. To overcome this impediment, we aimed to identify the entire spectrum of proteins involved in supporting survival after long-term incubation in water stress conditions. To this end, we used transposon insertion sequencing (Tn-seq) to identify mutants with low fitness in the presence of starvation in sterilized tap water (24).

Here, we identify proteins not previously known to support survival during water starvation (10, 14, 25), including a protein we show is critical for the infection of an amoebal host, as well as mammalian macrophages. Our study expands the current knowledge on the persistence factors of *L. pneumophila* and reveals new points of vulnerability for the organism during persistence in water.

## RESULTS

### Identification of *L. pneumophila* genes required for survival during starvation.

To identify candidate genes that are specifically essential for long-term survival of L. pneumophila in tap water but viable during growth in bacteriological medium, we employed mutagenesis with the mariner transposon Himar-1, which inserts randomly in chromosomal TA sites (26). From the pools generated, we used Tn-seq, in which massive parallel sequencing of transposon-chromosome junctions was performed to detect the locations of insertions before and after water starvation. We generated three independent mutant libraries with 64,000, 75,000, and 87,000 distinguishable members. This is equivalent to 26, 30, and 36% of the 248,087 theoretically possible integration sites, both within and outside annotated open reading frames (ORFs) (27). These three Tn-seq library data sets were then analyzed as a combined superpool representing a total of 54,491 mutants with distinct transposon insertions in ORFs, which represents 1/3 of the 166,297 theoretically possible ORF AT sites (NCBI SRA PRJNA816157).

**FIG 1:**
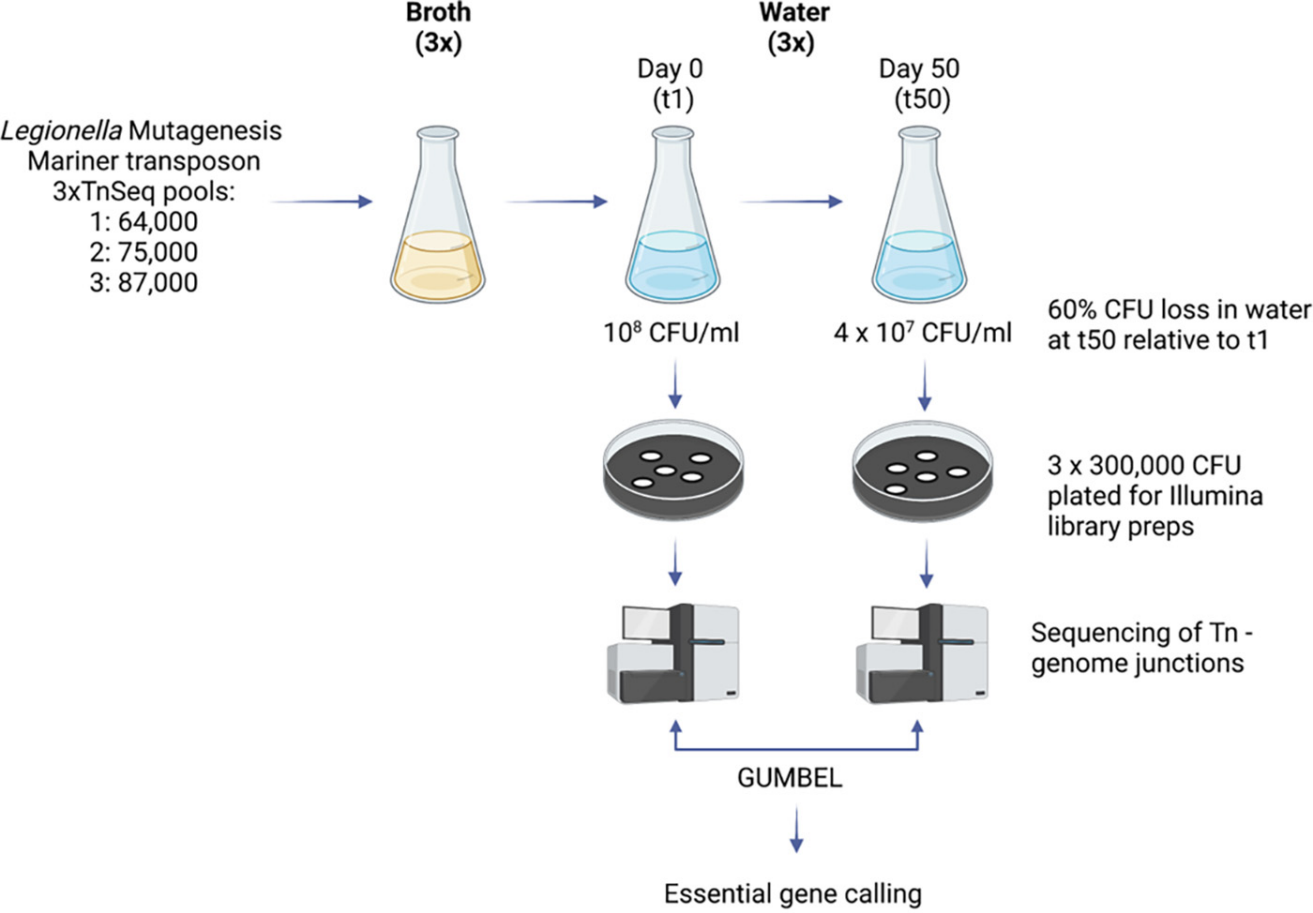
Tn-seq screening strategy for identification of L. pneumophila genes required for long-term survival in water. Three separate Tn-seq pools with 64,000 to 87,000 individual bacterial members were generated using the mariner transposon Himar-1. The individual pools were expanded to postexponential-growth phase in BYEα broth (OD_600_ 3.8) and then inoculated into sterilized tap water at an initial density of 10^8^ CFU/mL. After 50 days of incubation at 42°C, the CFU in water had dropped by 60% relative to t1. At t1 and t50, 300,000 CFU per library were plated and harvested for gDNA isolation and Illumina library preparation after 2 days of growth. Libraries were sequenced using Illumina HiSeq, and of transposon insertion frequencies per gene were determined at t1 and t50. The of insertion frequencies were subsequently statistically analyzed with GUMBEL to call essential genes at the t1 and t50 (24). The figure was created with BioRender.com.

The mutant libraries were subjected to water stress at 42°C, taking advantage of the fact that elevated temperatures are known to accelerate viability decay in water, shortening the time required to analyze the data sets (9, 28) (Fig. 1). To this end, 300,000 CFU (the CFU for each library) were incubated on solid bacteriological medium and harvested either directly after tap water inoculation (t1) or after 50 days water exposure (t50). The CFU/mL in water of each library dropped by 60% during the 50-day incubation (from 1 × 10^8^ to 4 × 10^7^ CFU/mL). Genes specifically necessary for high fitness under starvation conditions were discovered by using the Bayesian/GUMBEL method embedded in the TRANSIT software package (24), identifying mutants that were poorly recovered at 50 days relative to their abundance on initial inoculation into water (Fig. 1). This approach identifies ORFs with long stretches showing much lower insertion density than observed prior to water incubation. The probability of such insertion gaps occurring by chance is then calculated by a Bayesian analysis of the GUMBEL distribution, and essential (poor representation relative to initiation of incubation), nonessential, and “uncertain” or equivocal phenotypes are called (24).

**FIG 2:**
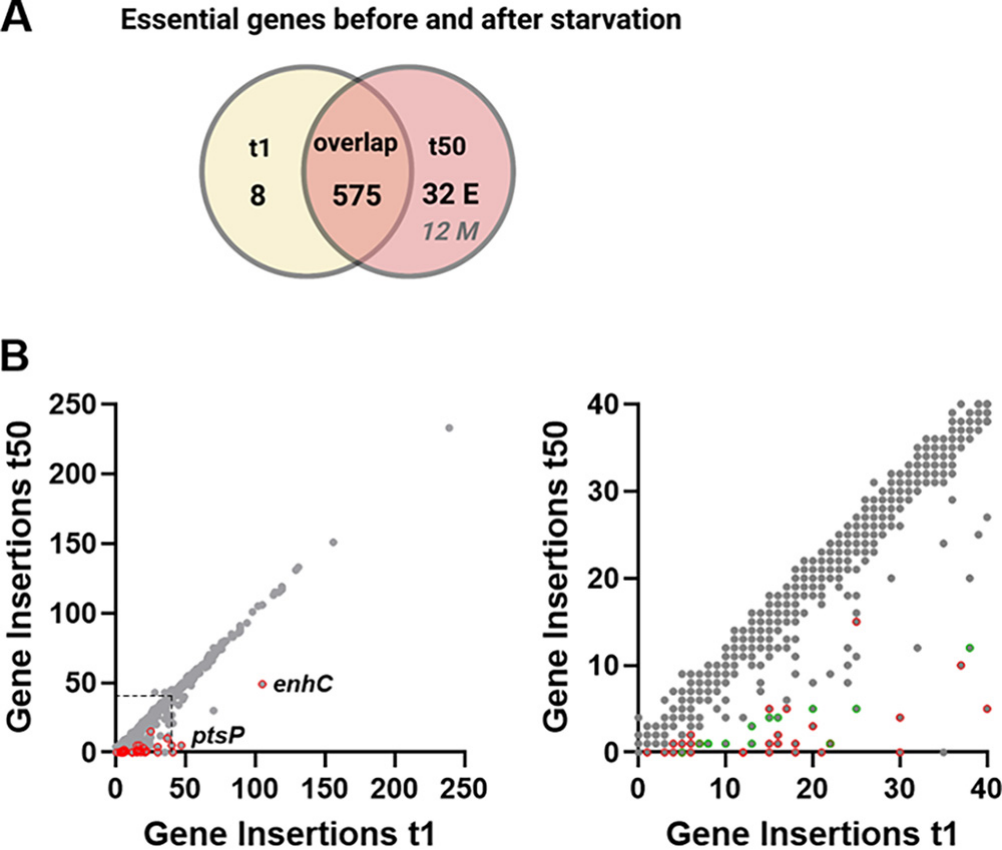
Strategy for identification of 44 genes critical for survival during long term water stress. Identification of essential genes after 50 days starvation in water at 42°C compared to bacteriological medium followed by short water exposure. (A) GUMBEL analysis identified 575 genes essential (labeled E) both after immediate addition into water (t1, yellow circle) or incubating for 50 days at 42°C (t2, red circle; overlap). By manual selection (labeled M), we identified 12 additional genes presumably important for starvation survival in t50 that where below the threshold of significance in GUMBEL (italic number, labeled M). (B) Comparison of insertion abundance/gene for bacteria at t50 versus t1. The left diagram shows all genes (gray) with the 32 essential genes called by GUMBEL encircled in red. The right diagram shows the 0 to 40 insertions/gene region only (boxed on the left side). The red circles indicate essential genes under water starvation conditions called by GUMBEL. The green circles indicate additional genes presumably important for starvation survival based on low abundance after 50-day water exposure. Members of the additional gene set had a minimum of five insertions at t1. After starvation, the abundance of insertions occurring in members of the latter group was reduced by >3-fold (12 genes). Figure 2A was created with BioRender.com.

Based on the analysis of the Tn-seq superpool, of 2,943 annotated L. pneumophila Philadelphia 1 ORFs (NC002942), 583 genes were called essential (E) after growth in bacteriological media (24) (Fig. 2A; see also Table S1 in the supplemental material). When this pool was subjected to water stress for 50 days, the essential set included 32 additional genes (Fig. 2B, red). We also included genes with a >3-fold drop in abundance at t50 relative to t1 in our analysis to account for genes with severely reduced fitness after the treatment but did not reach the threshold of significance at the employed library saturation level (Fig. 2B, green data points; see also Table S1 in the supplemental material). The combined gene sets resulted in a list of 44 genes (Table 1), which we defined as the candidate gene set required for survival after prolonged starvation of L. pneumophila in water. The candidate genes were ordered according to their known or predicted functions and overrepresented in functions associated with the bacterial envelope (Table 1). These included factors involved in respiration (*n* = 12 genes); membrane transport (*n* = 8); hypothetical proteins (*n* = 5); regulation (*n* = 6); “virulence” (*n* = 3); DNA replication, repair, and recombination (*n* = 3); intermediary metabolism (*n* = 5); stress (*n* = 1); and protein processing (*n* = 1) necessary for high viability under water stress conditions. Of particular note is the reidentification of *rpoS* sigma factor and the *letAS* regulatory system as being essential under water starvation conditions. Since these three genes had been previously discovered as controlling survival under water starvation, this argues that our selection procedure reproduces previously established starvation conditions in the literature (14, 25).

**TABLE 1:** Candidate gene set required for survival after prolonged starvation in water^*a*^

Gene function and designation	Name	t1	Call t1	t50	Call t50	Description	CRISPRi
Respiration							
lpg0011	*resA*	4	NE	0	E	Thiol-disulfide oxidoreductase	
lpg0412	*lpg0412*	12	NE	0	E	Protoheme IX famesyltransferase	
lpg0857	*ccmB*	5	NE	1	E	Heme exporter	
lpg0860	*ccmE*	4	NE	1	E	Cytochrome *c* biogenesis	
lpg0864	*cycH*	6	NE	2	E	Cytochrome *c* type biogenesis	
lpg2703	*lpg2703*	15	NE	1	E	Ubiquinol-cytochrome *c* reductase, cytochrome *c*_1_	
lpg2705	*petA*	6	NE	0	E	Ubiquinol-cytochrome *c* reductase, Fe-S subunit	
lpg2896	*lpg2896*	30	NE	0	E	Cytochrome *c* oxidase subunit I CtaD	
lpg2897	*lpg2897*	18	NE	0	E	Cytochrome *c* oxidase subunit II	
lpg2898	*lpg2898*	21	NE	0	E	Cytochrome *c*	
lpg2894	*cycC*	5	U	0	E	Cytochrome *c* oxidase subunit 3	
lpg2704	*petB*	25	NE	5	U	Ubiquinol-cytochrome *c* reductase, cytochrome *b*	
Transport							
lpg1360	*lspI*	3	NE	0	E	Type II secretory pathway protein LspI	
lpg2045	*mlaD*	16	NE	2	E	ABC transporter substrate-binding protein	
lpg2046	*mlaF*	16	NE	1	E	ABC transporter ATP-binding protein	
lpg2047	*mlaE*	22	NE	1	E	ABC transporter permease	
lpg2871	*ptsP*	47	NE	5	E	Phosphoenolpyruvate protein phosphotransferase	
lpg0895	*lspC*	13	NE	3	NE	Type II secretion system protein C	
lpg2930	*tatC*	15	NE	4	NE	Sec-independent protein translocase protein (TatC)	
lpg2044	*pqiC*	22	NE	1	U	ABC-type intermembrane transport lipoprotein	
Hypothetical							
lpg0292	*lpg0292*	18	NE	1	E	Hypothetical protein	
lpg0410	*lpg0410*	6	NE	1	E	Hypothetical protein	
lpg1697	*lpg1697*	15	NE	0	E	Hypothetical protein	
lpg1859	*lon*	30	NE	4	E	Hypothetical protein Lon superfamily	
lpg2969	*lpg2969*	22	NE	1	E	Hypothetical protein ElaC superfamily	
Regulation							
lpg0726	*nrdR*	1	NE	0	E	Transcriptional regulator NrdR	
lpg1284	*rpoS*	5	NE	0	E	Stationary-phase-specific sigma factor RpoS	
lpg1521	*lpg1521*	12	NE	0	E	Methyltransferase, class I SAM dependent	
lpg1912	*letS*	41	NE	0	E	Sensory box histidine kinase/response regulator	
lpg0537	*letE*	7	NE	1	U	Transmission trait enhancer LetE	
lpg2646	*letA*	10	NE	1	U	Response regulator LetA	
Metabolism							
lpg0805	*ppsA*	40	NE	5	E	Phosphoenolpyruvate synthase	
lpg1350	*lpg1350*	20	NE	3	E	l-Lysine dehydrogenase	
lpg0639	*deoB*	8	U	1	E	Phosphopentomutase	
lpg1584	*pcsA/lidK*	20	NE	5	NE	Phosphatidylcholine synthase	
lpg0102	*fabF*	16	NE	4	NE	3-Oxoacyl-(acyl carrier protein) synthase	
DNA replication, repair, and recombination							
lpg0072	*uvrB*	37	NE	10	E	Excinuclease ABC subunit B	
lpg1812	*lpg1812*	25	NE	15	E	UvrD/Rep helicase	
lpg2645	*uvrC*	38	NE	12	U	Excinuclease ABC subunit C	
Virulence							
lpg2639	*enhC*	105	NE	49	E	Enhanced entry protein EnhC	
lpg2640	*enhB*	17	NE	5	E	Enhanced entry protein EnhB	
lpg0791	*mip*	15	NE	5	E	Macrophage infectivity potentiator Mip	
Protein processing							
lpg0686	*dsbD*	13	U	1	E	Disulfide interchange protein DsbD	
Stress							
lpg0935	*lpg0935*	7	NE	1	E	Universal stress protein A UspA	

a t1 and t50, number of insertions per gene observed at time point; Call t1/t50: E, essential; NE, nonessential; U, uncertain. CRISPRi, gray-shaded tiles indicate genes chosen for validation by knockdown. Manually identified genes are separated by dashed line at the end of each section (see Fig. 2B). Further details are provided in Table S1.

10.1128/msphere.00454-22.3 TABLE S1 GUMBEL analysis of gene essentiality. Download Table S1, XLSX file, 0.3 MB. Copyright © 2023 Aurass et al. 2023 Aurass et al. https://creativecommons.org/licenses/by/4.0/ This content is distributed under the terms of the Creative Commons Attribution 4.0 International license.

### Efficient knockdown of gene transcription with CRISPR interference.

We next directed our analyses to individual members of the candidate list of water stress survival genes, focusing on proteins not previously noted as important for survival in water starvation conditions (Table 1), to validate the predictions made by our Tn-seq analysis. For this purpose, we took advantage of the recently established CRISPR interference system for L. pneumophila (29). This system utilizes an anhydrotetracycline (ATC)-inducible *dcas9* gene inserted in the nonfunctional chromosomal *thyA* locus of *L. pneumophila* strain Lp02, and crRNA, as well as tracrRNA-encoding sequences on a plasmid (29, 30).

**FIG 3:**
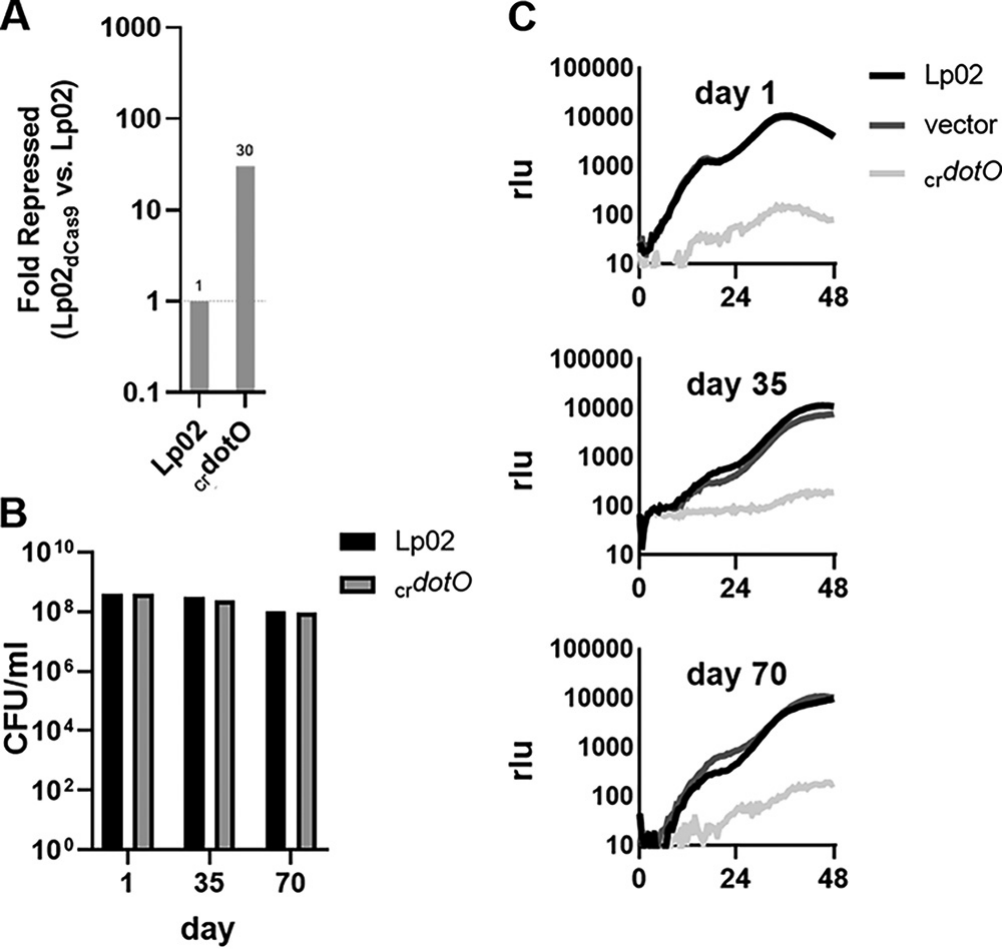
CRISPRi strategy results in efficient depletion and allows blockade of intracellular replication under starvation conditions. (A) Reduction of steady-state transcript levels by CRISPRi after growth in broth and inoculation in water (t1). Lp02dCas9Lux(_cr_*dotO*) was grown in the presence of ATC inducer in broth and inoculated in water, and transcription levels were compared to Lp02 (WT) by qRT-PCR. (B) High viability of Lp02Lux and _cr_*dotO* strains during extensive water exposure. Viability was measured by spot plating CFU/mL on BCYEα agar. (C) CRISPR interference of *dotO* blocks intracellular growth. Bacteria were inoculated in tap water for the noted times and then used to challenge A. castellanii over 72 h. Vector, Lp02dCas9Lux (pMME1936). Intracellular growth was measured based on the luminescence. The multiplicity of infection used was 0.5. rlu, relative light units.

Previous work has demonstrated that CRISPRi technology allows efficient gene knockdowns in L. pneumophila during growth in either broth or phagocytic cells (29). Its applicability to prolonged water starvation conditions has not been previously established. Therefore, we tested a knockdown in a gene encoding an Icm/Dot component, which has no effect on long-term survival in water (NCBI SRA PRJNA816157; see Table S1) but blocks intracellular growth, to determine the effectiveness of this strategy. This allowed us to determine whether long-term water incubation altered the efficiency of ATC-activated CRISPRi depletion. To this end, a _cr_*dotO* strain (CRISPRi-*dotO*), which showed a 30-fold knockdown efficiency relative to control Lp02 when assayed after growth to postexponential (PE) phase, was tested for its ability to block growth within Acanthamoeba castellanii after water starvation (Fig. 3A). To minimize the adverse effects on bacterial viability but maximize the timescale we performed this experiment at ambient temperature (22°C), starving for either 35 or 70 days (Fig. 3).

The effects of the *dotO* CRISPRi knockdown were monitored phenotypically, since gene expression analysis in starved bacteria is made difficult by the large-scale drop in transcription and changes in gene expression dynamics that occur under these conditions relative to broth-grown bacteria (31). After growth in broth to postexponential phase, there was a 30-fold drop in steady-state levels of *dotO* transcript in the presence of *dotO* crRNA (Fig. 3A). As predicted by the Tn-seq data set, this caused no reduction in viability relative to the wild type (WT) at either 35 or 70 days of tap water starvation (Fig. 3B). In contrast, *dotO* CRISPRi knockdown resulted in a total block in growth within A. castellanii of *L. pneumophila* harboring a luciferase reporter, with the level of defect indistinguishable for bacteria undergoing long-term water starvation (35 or 70 days) or for bacteria assayed shortly after removal from broth and washing in water (Fig. 3C). These data demonstrate that we could separate water survival from defective intracellular growth and that CRISPRi knockdown persists over long-term water incubation, allowing phenotypic assays to be used in lieu of a direct measure of transcription after starvation. We conclude that the CRISPRi system (29), in addition to efficient gene knockdown in broth-grown *L. pneumophila*, can be used to evaluate starvation survival and infection dynamics after long-term water stress.

### Demonstration that candidates are required for survival under water stress conditions.

Having demonstrated the combined applicability of CRISPRi and the bacterial luciferase operon as an indicator of intracellular survival, we next created single crRNA-encoding plasmids for specific candidate water stress survival genes as well as a nontargeting sequence derived from the mRFP ORF. These were then introduced into the WT strain Lp02dCas9Lux (32) (Table 1; see also Table S2). This strain has the bacterial luciferase operon *luxCDABE* downstream of the chromosomal P*ahpC* in the *thyA*::dCas9 background (33, 34), allowing the downstream analysis of metabolic activity of CRISPRi depletion strains. The luciferase system uses cofactors, such as NADPH, ATP, FMNH2, and acetyl coenzyme A, allowing it serve as an intrinsic proxy for metabolic activity (33, 35). The observed loss of luciferase activity is likely due to the loss of metabolic activity, so the absence of activity after metabolic stimulation by laboratory growth media or after coculture with permissive host cells is a signal that the bacteria are presumably dead.

10.1128/msphere.00454-22.4 TABLE S2 Oligonucleotide list. Download Table S2, XLSX file, 0.01 MB. Copyright © 2023 Aurass et al. 2023 Aurass et al. https://creativecommons.org/licenses/by/4.0/ This content is distributed under the terms of the Creative Commons Attribution 4.0 International license.

Since our Tn-seq experiment relied on the ability of a mutant to compete against a large population, we wanted to test whether the candidates simply showed poor competition but otherwise were fit when incubated in isolation during long-term water starvation. To address this point, individual crRNA derivatives were grown in broth in the presence of ATC and then subjected to water starvation as described for the *dotO* depletion. Analysis was directed toward 18 candidates hypothesized to be involved in water stress survival, picking representative members of each functional group, as well as the known water stress survival gene *rpoS* as a control. These were chosen to avoid independent testing of multiple genes belonging to the same functional group, such as the respiratory chain genes (Table 1) (25), and already characterized stress survival genes such as *letS* and *uspA*.

**FIG 4:**
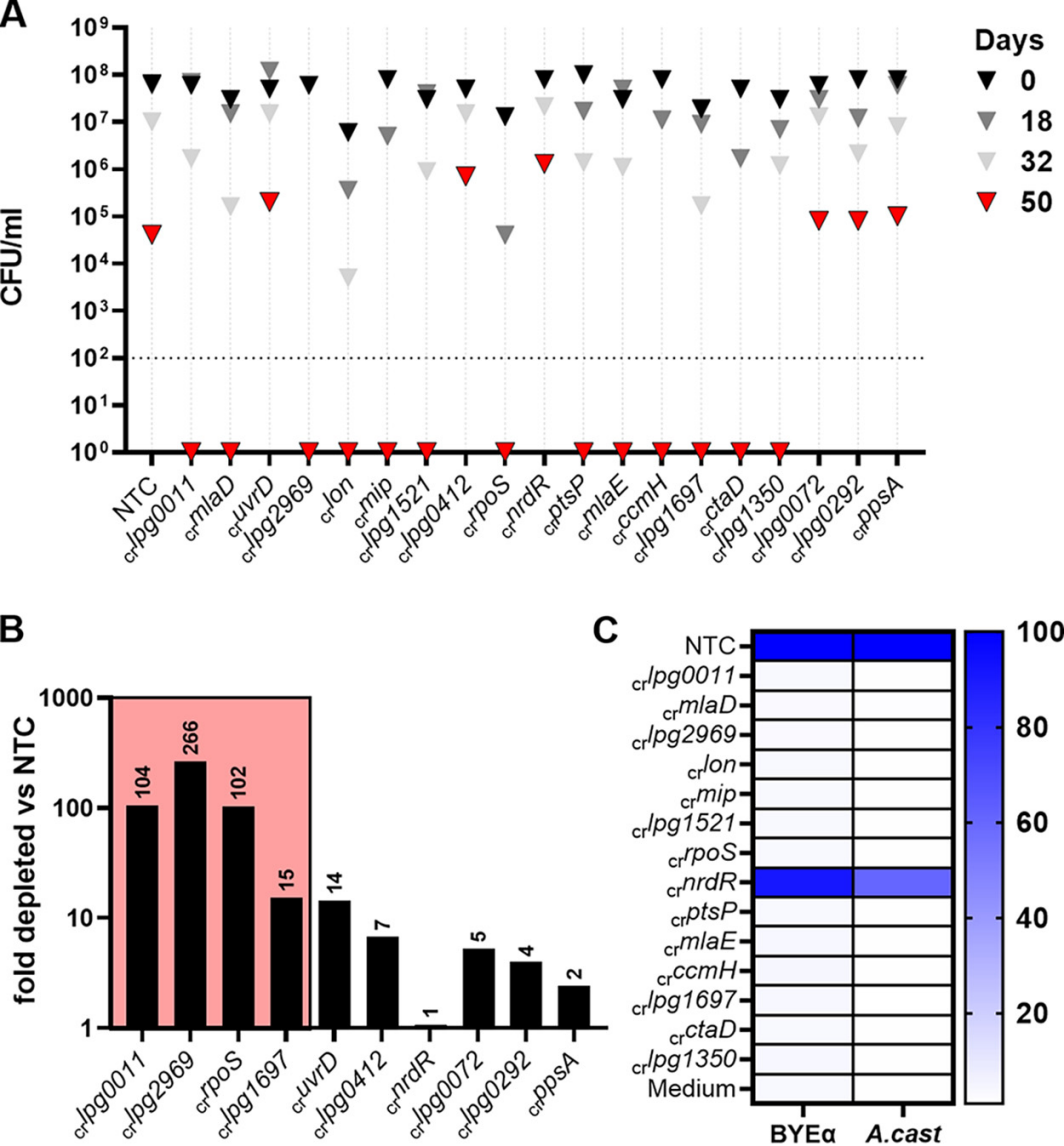
CRISPRi knockdown results in loss of viability of water stress survival-defective candidates after long-term water exposure. (A) CFU/mL of 19 candidate CRISPRi knockdown strains identified as conditionally essential by Tn-seq after extensive water stress. Dotted line, detection limit. Triangles: black, 0 days; dark gray, 18 days; light gray, 32 days; and red, 50 days. Each strain represents a single clonal water culture. (B) Efficiency of CRISPRi depletion correlates with stress survival phenotype. Analysis of knockdown efficiency in select knockdown strains relative to the nontargeting control. Strains were chosen based on culture phenotype, including four culture-undetectable strains after water incubation (colored box) and the six strains that still formed colonies after 50 days water incubation. (C) Culture in *A. castellanii* does not rescue CRISPRi candidates that are not viable in bacteriological medium. A heat map of the RLU expressed as the area under curve after 24 h (BYEa) or 72 h (*A. cast*) of growth in BYEa or during coculture in A. castellanii of culture-negative strains, as well as NTC and _cr_*nrdR* culture-viable controls, is shown.

Bacteria were incubated in tap water at 42°C, and at various times over a 50-day period, aliquots were serially diluted onto solid bacteriological medium to quantitate viable bacteria, comparing CFU to the nontargeting control (Fig. 4A). Water incubation for 18 days resulted in a number of the candidates showing a loss of viability relative to the nontargeting control (Fig. 4A, charcoal triangles). Most striking was crRNA directed against *lpg2969*, which showed no detectable CFU after 18 days of incubation (Fig. 4A; 10^−7^ CFU relative to nontargeting control). Water starvation conditions for 32 days resulted in decreased viability of 67% of the strains that were tested, with depletions of *mip*, *rpoS*, *ccmH*, and *ctaD* also showing no viability at this time point, with extremely poor viability of the *lon*, *lpg1697*, and *mlaD* depletions. After 50 days in water, depletions of the additional candidate genes *lpg0011*, *mlaD*, *lon*, *lpg1521*, *ptsP*, *mlaE*, *lpg1697*, and *lpg1350* showed no viability on plates. Since the majority (68%) of the depletion derivates resulted in the absence of viability in water, our selection procedure identified mutants that are involved in tolerating water starvation conditions even in the absence of competition with the whole populations (Table 2).

**TABLE 2:** Essential starvation survival genes identified by Tn-seq and confirmed by CRISPRi knockdown

Gene	Name	CD^*a*^	Localization^*b*^	Function^*b*^	PMID no.
*lpg0011*	Thiol-disulfide oxidoreductase	TlpA_like_family	Periplasm	Energy metabolism	
*lpg0791/mip*	Macrophage infectivity potentiator	FkpA/COG0545	Membrane	Pathogen	1594630
*lpg0864/ccmH*	Cytochrome *c* type biogenesis protein ccmh	NrfG super family	Periplasm	Energy metabolism	
*lpg1284/rpoS*	Stationary-phase-specific sigma factor	rpoS_proteo/TIGR02394	Cytosolic	Regulation	25416763
*lpg1350*	l-Lysine dehydrogenase	Lys9 super family	Cytosolic	Lysine metabolism	
*lpg1521*	Methyltransferase	AdoMet_MTases super family	Cytosolic	Regulation/modification	
*lpg1697*	Hypothetical protein	YkuD_2/pfam13645	Periplasm	Peptidoglycan modeling	
*lpg1859/lon*	ATP-dependent protease La	Lon/COG0466	Cytosolic	Protein homeostasis	17891379
*lpg2045/mlaD*	ABC transporter substrate-binding protein	MlaD/COG1463	Inner membrane	Membrane homeostasis	
*lpg2047/mlaE*	ABC transporter permease	MlaE/STAS superfamily	Transmembrane	Membrane homeostasis	
*lpg2871/ptsP*	Phosphoenolpyruvate protein phosphotransferase	PtsP super family/cl34645	Inner membrane	Energy metabolism	
*lpg2896/ctaD*	Cytochrome *c* oxidase subunit I	Cyt_c_Oxidase_I/cd01663	Inner membrane	Energy metabolism	
*lpg2969*	Hypothetical protein	ElaC/COG1234	Cytosolic	Translation	

a Source: NCBI CDD database. Entries are indicated as “name/accession number” where applicable.

b Known or inferred from CDD.

Of the 19 candidates chosen for validation by CRISPRi, 6 resulted in approximately the same fraction of survivors after 50 days starvation in water as the nontargeting control. To test whether absence of phenotypes in these strains could be due to insufficient target depletion by dCas9, we performed qRT-PCR with RNA isolated from the culture aliquots obtained at the onset of water cultures after growth in broth. In addition, we chose four confirmed candidate genes and the NTC for reference (Fig. 4B). Transcript depletion of the confirmed genes was 104-fold (*lpg0011*), 266-fold (*lpg2969*), 104-fold (*rpoS*), and 15-fold (*lpg1697*). In contrast, the depletion levels of the targeted transcripts from the strains showing no apparent survival defect were lower, ranging from 14-fold (_cr_*uvrD*) to no effect (_cr_*nrdR*) (Fig. 4B). These data are consistent with inefficient transcript depletion being the cause of the absence of detectable phenotypes in these strains.

We next determined whether the strains showing low viability on solid medium were metabolically inactive based on loss of luciferase activity and restriction by A. castellanii. After 50 days of starvation, culture aliquots of the 13 strains that showed no viability on solid medium at this time point were metabolically stimulated and cocultured with A. castellanii in an attempt to resuscitate unculturable L. pneumophila (Fig. 3C). The control strains (NTC and the _cr_*nrdR*-harboring strains) readily responded to metabolic stimulation with increased luciferase activity and were capable of intracellular growth (Fig. 3C). In contrast, there was no evidence of luciferase activity in the strains tested, and none of these strains were resuscitated by coculture with amoebae. Since no signs of metabolic stimulation through nutrient addition or coculture were evident in the knockdown strains (Fig. 3C), we conclude that knockdown of this particular set of genes resulted in no evidence of resuscitation under any condition, and their loss caused bacterial cell death.

We conclude that, as predicted by the results of the original selection, overrepresented processes such as maintenance of bacterial envelope homeostasis (*mip*, *lpg1697*, and *mlaDEF*), electron transport (*ctaD*), and cytoplasmic protein turnover (*lon*) are critical components in supporting survival in the face of water stress (Table 2). The consequence of loss of these proteins, which are not essential during growth in bacteriological medium, is bacterial death after 50 days of incubation in tap water.

### Lpg1697 carrying a YkuD domain is required for survival in the presence of water stress.

*Lpg1697* encodes a predicted 225-amino-acid polypeptide carrying a predicted l,d-transpeptidase domain (LDT; YkuD_2) and a predicted N-terminal signal peptide (SignalP-5.0). Previous studies showed the peptidoglycan layer of *L. pneumophila* is highly cross-linked (36), and as many as 10 LDTs have been predicted to be encoded by the genome of L. pneumophila (37) (see Fig. S1). Unlike characterized LDTs of other genera, Lpg1697 lacks a predicted LysM peptidoglycan-binding domain, questioning its functionality as a murein-modifying enzyme. In fact, canonical LysM/YkuD LDTs are absent in the L. pneumophila genome, so we investigated its potential function in maintaining cell wall integrity.

10.1128/msphere.00454-22.1 FIG S1 Partial ClustalOmega amino acid sequence alignment of YkuD like domain proteins of *L. pneumophila*. Conserved residues involved in catalysis are boxed red. Download FIG S1, TIF file, 0.4 MB. Copyright © 2023 Aurass et al. 2023 Aurass et al. https://creativecommons.org/licenses/by/4.0/ This content is distributed under the terms of the Creative Commons Attribution 4.0 International license.

**FIG 5:**
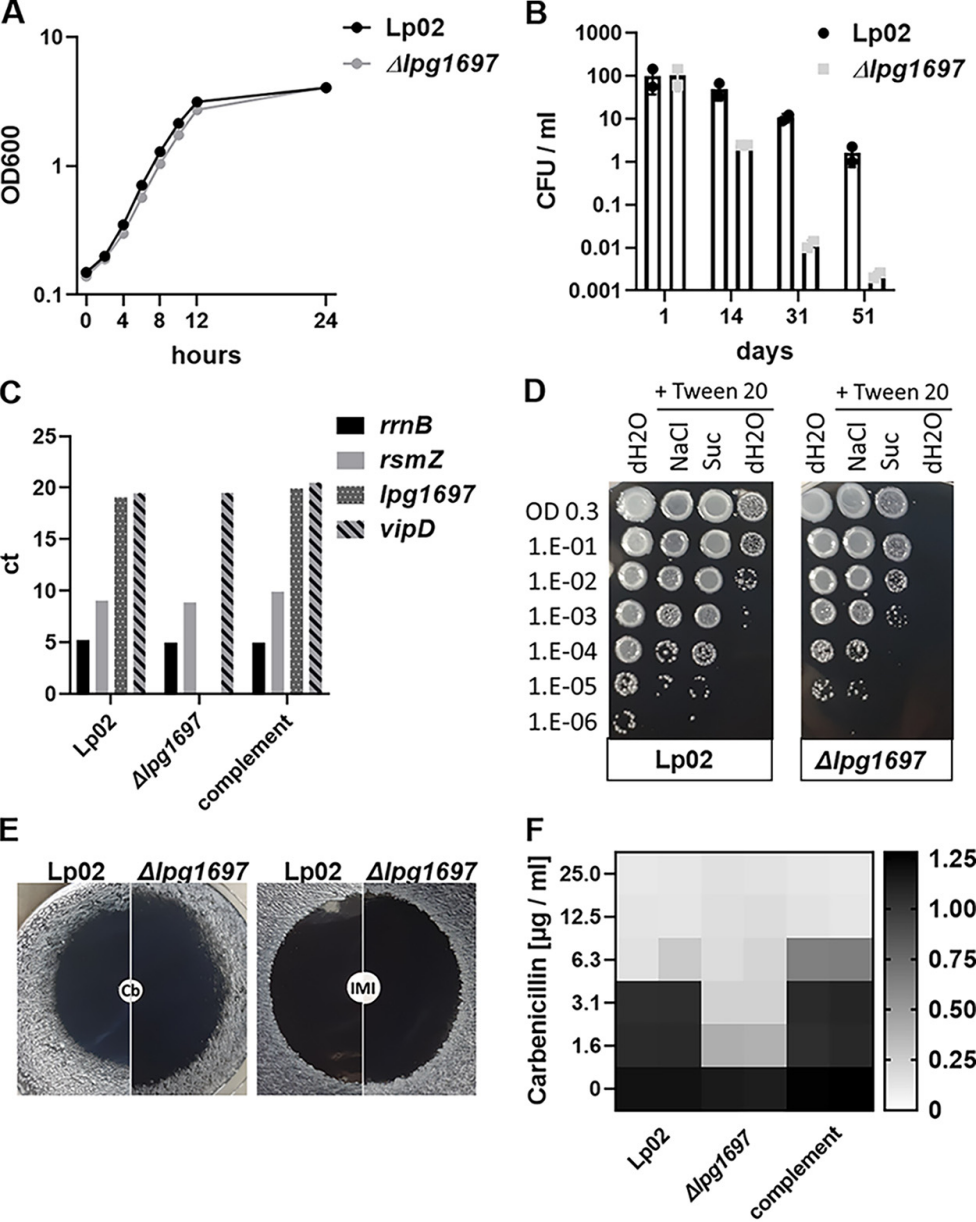
Loss of Lpg1697 results in defective survival in water, as well as hypersensitivity to carbenicillin and osmotic stress. (A) The *Δlpg1697* strain shows no fitness defect during broth growth. (B) Water stress results in decreased viability of the L. pneumophila *Δlpg1697* strain relative to the WT. (C) qRT-PCR analysis of four genes in the noted stains. The L. pneumophila *Δlpg1697* strain shows no detectable transcription of *lpg1697.* The transcript levels of *rsmZ* are similar in all strains tested. Complement, L. pneumophila *Δlpg1697* carrying *lpg1697* in a neutral genomic site (38). (D) *Δlpg1697* mutants are hypersensitive to Tween 20. The addition of osmostabilizers suppressed this effect. (E) The *Δlpg1697* mutant is hypersensitive to carbenicillin. Disc diffusion assays were performed with carbenicillin (left) and imipenem (right), as described in Materials and Methods. (F) The *Δlpg1697* mutant has a lower MIC to carbenicillin relative to the WT. The *A*_600_ was determined after 46 h of growth measured in a 96-well plate.

An in-frame deletion mutation was constructed (*Δlpg1697*) and tested for growth in broth culture and survival after prolonged water exposure. Growth in broth was indistinguishable for the deletion and the WT (Fig. 5A); however, after 51 days of incubation in water at 42°C, the survival of the mutant was <10^3^ that of the WT (Fig. 5B). Furthermore, the mutant clearly lost viability relative to the WT by 14 days postincubation. The *lpg1697* gene is located directly adjacent to the *rsmZ* noncoding RNA in L. pneumophila. To exclude polar effects of *lpg1697* deletion on *rsmZ* expression, we analyzed the expression of *lpg1697* and *rsmZ*, as well as the unlinked *vipD* and *rrnB* genes, as controls after growth in broth (Fig. 5C). With the exception of *lpg1697*, the expression of each gene in the mutant background was comparable to the WT (Fig. 5C). Transcription was restored by complementation of *lpg1697* in the chromosome (38) (Fig. 5C).

Given the presence of a predicted LDT domain in Lpg1697, we next tested deletion mutants for cell envelope phenotypes by exposure to envelope stress and to cell wall-targeting antibiotics. After growth in broth to PE phase and 30 min of treatment with 0.05% Tween 20 in ultrapure water, the CFU of *Δlpg1697* strain decreased by 10^3^ compared to the WT (Fig. 5D). The addition of salt (300 mM NaCl) or sucrose (300 mM) to the 0.05% Tween 20 solution suppressed the effects of the detergent/hypotonic solution. NaCl at 300 mM was particularly effective, with viability restored to that of the WT, consistent with a loss of Lpg1697 leading to a loss of cell envelope integrity.

LDTs in other organisms are resistant to most carboxypenicillins such as Cb, but can be inhibited by carbapenems, such as imipenem (39–41). To further discriminate Lpg1697 function, we performed a zone of inhibition assay, comparing sensitivity to the carbapenem antibiotic imipenem (Fig. 5E). The deletion mutant was hypersensitive to Cb, showing an increased zone of inhibition relative to the WT, whereas in the presence of imipenem, there was no distinguishable difference between the mutant and the WT (Fig. 5E). Concordantly, the carbenicillin MIC decreased 4-fold in *Δlpg1697* strains but was comparable to the WT in the complementing strain (Fig. 5F). Therefore, the loss of Lpg1697 results in a clearly definable loss in resistance to an antibiotic that shows higher efficacy against other known penicillin binding proteins, consistent with Lpg1697 having LDT activity.

We therefore next analyzed murein composition of the WT and deletion strains by ultrahigh-performance liquid chromatography (UHPLC) (42). PE-phase *L. pneumophila* was used because, growth under these conditions, it resulted in enhanced antibiotic and osmotic-challenge hypersensitivity of the mutant (Fig. 5D to F). Surprisingly, the murein profile of the *Δlpg1697* strain resembled the WT profile without detectable changes in either muropeptide patterns, the fraction of l,d-cross-links, and overall cross-linking (see Fig. S2). This is consistent with the absence of Lpg1697 causing subtle differences in peptidoglycan structure that result in envelope instability that are difficult to detect using this particular strategy.

10.1128/msphere.00454-22.2 FIG S2 Absence of detectable changes in muropeptide profiles related to loss of *lpg1697* gene. (A) Chromatograms of UHPLC runs. (B) Relative amounts of total cross-linkages and l,d-cross-linkages were not significantly different between the strains (two tailed unpaired *t* test). Download FIG S2, TIF file, 0.2 MB. Copyright © 2023 Aurass et al. 2023 Aurass et al. https://creativecommons.org/licenses/by/4.0/ This content is distributed under the terms of the Creative Commons Attribution 4.0 International license.

### Loss of Lpg1697 function results in defects in intracellular growth.

**FIG 6:**
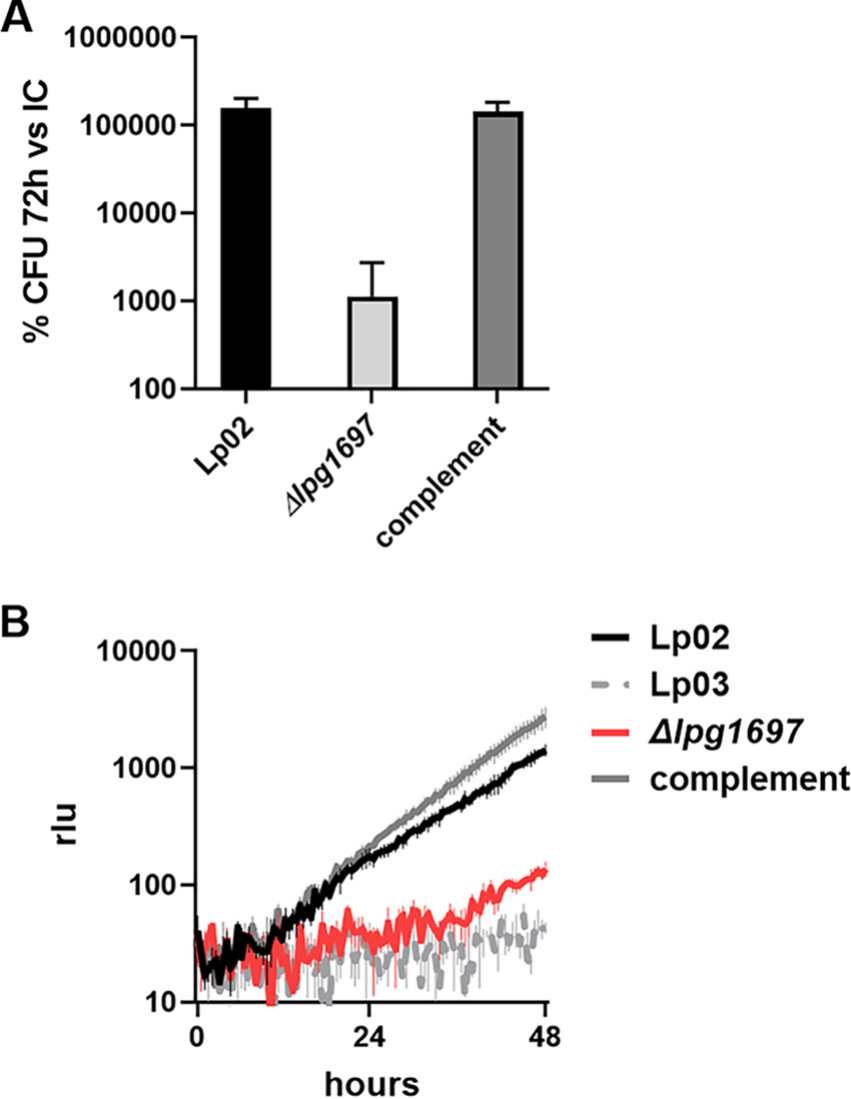
Lpg1697 function is required for efficient intracellular growth in A. castellanii and macrophages. (A) Impaired growth of the *Δlpg1697* strain within A. castellanii. Growth was assayed by determining the CFU on CYE plates at 72 h postinfection. Complement, L. pneumophila *Δlpg1697* strain carrying *lpg1697* in a neutral genomic site (38). (B) Impaired intracellular growth of the *L. pneumophila Δlpg1697* strain and a type 4 secretion system mutant (LP03) in BMDMs and restoration of growth by the complementing strain (MOI = 0.01).

Having established that loss of *lpg1697* negatively impacts the capability of *L. pneumophila* to persist during water stress, we were interested whether its loss also reduces intracellular growth fitness after culturing to the PE phase under permissive conditions. To this end, the WT and an isogenic type 4 secretion system mutant (Lp03) were compared to the *Δlpg1697* strain by challenging both A. castellanii and murine bone marrow derived macrophages (BMDMs) at MOIs of 0.05 and 0.01, respectively (Fig. 6). Strikingly, intracellular growth of the Δ*lpg1697* mutant was severely attenuated in amoebae with 100-fold lower CFU at 72 h postinfection compared to the WT or the strain harboring the complementing gene (Fig. 6A). There was a similar level of attenuation of intracellular growth during BMDM challenge (Fig. 6B), indicating the Lpg1697 is required for high persistence in water, as well as high fitness after uptake into host cells.

Taken together, we confirmed importance of 12 previously unrecognized water stress-related genes of L. pneumophila. Our data highlight an essential role of a YkuD domain protein for persistence and intracellular growth in A. castellanii and macrophages. To our knowledge, this is the first report highlighting conditional essentiality of a YkuD domain protein in *Legionella* without an apparent link to peptidoglycan structure.

## DISCUSSION

As a waterborne pathogen, L. pneumophila transmission to humans is tightly linked to its survival in tap water. Since *L. pneumophila* has complex nutritional requirements, including auxotrophies for at least eight amino acids (43), it cannot proliferate in the absence of appropriate nutrients or a permissive host. In spite of these restrictions, it is capable of persisting for more than a year in water (44–46). We provide here a comprehensive screen for genes that are essential for this process. Tn-seq analysis revealed 44 genes whose interruptions were not tolerated by starving L. pneumophila. Using CRISPR interference, we showed that a large number of these genes are essential for survival in clonal populations, while the remaining genes might constitute fitness factors with an essential role only under competitive conditions. The *Legionella* genus exhibits high genetic versatility (47), and pan-genus conservation of proteins is indicative of physiological importance. To analyze conservation of the 44 candidate starvation factors across the *Legionella* genus, we used the comprehensive ortholog table of Gomez-Valero et al. (47) and analyzed protein conservation across 79 different *Legionella* species and *L. pneumophila* strains. Notably, the overall degree of conservation is high with orthologs of 31 candidates in all 79, 10 candidates in 78, two candidates in 77, and 1 in 62 different species and *L. pneumophila* strains (see Table S4). Most notably, almost all of the most commonly used *L. pneumophila* genomes (strains Paris, Corby, and Lens) and the soil thriving species *L. longbeachae* encode orthologs of all 44 candidate proteins (see Table S5). Strain 130b encodes 42 orthologs. Our data extend the identification of Achilles heel genes required under water stress conditions, uncovering novel vulnerabilities of the pathogen which, based on orthology analysis, appear to be almost universal within the *Legionella* genus.

10.1128/msphere.00454-22.6 TABLE S4 Presence/absence ortholog table. Download Table S4, XLSX file, 0.02 MB. Copyright © 2023 Aurass et al. 2023 Aurass et al. https://creativecommons.org/licenses/by/4.0/ This content is distributed under the terms of the Creative Commons Attribution 4.0 International license.

10.1128/msphere.00454-22.7 TABLE S5 Ortholog table across most commonly used *L. pneumophila* strains and *L. longbeachae*. Download Table S5, XLSX file, 0.01 MB. Copyright © 2023 Aurass et al. 2023 Aurass et al. https://creativecommons.org/licenses/by/4.0/ This content is distributed under the terms of the Creative Commons Attribution 4.0 International license.

Previous studies indicated the CsrR regulator is an important persistence factor for *L. pneumophila* and a reciprocal model in which CsrR governs *L. pneumophila* environmental resilience, while its paralog repressor, CsrA (12), that directs its intracellular replication-transmission cycle has been proposed (17). In addition, an RpoS-dependent operon *lpg0279-77* encoding a two-component signaling system and a putative regulator have been reported to support survival in a nutrient-scarce environment (48). Our transposon analysis could not evaluate the importance of CsrR during water starvation, since the number of AT targets was too small to make an accurate call. On the other hand, the RpoS-regulated operon appeared nonessential in either broth or water starvation conditions (see Table S1). Similarly, LasM and a putative transcriptional regulator *lpg2524* both promote survival of *L. pneumophila* in artificial freshwater (18, 19), but we could find no evidence for fitness defects in tap water (see Data Set S1). Other previous work concluded a role for the stringent response and RpoS, as well as the LetA/S two-component signaling systems for successful persistence in water by orchestration of a broad transcriptomic downshift concurrent with the activation of transmissive traits (14, 25, 49). Both the *rpoS* and the *letAS* genes were considered essential for long-term water survival based on our Tn-seq analysis, with no survival of these mutants after 50 days in water. Similarly, we found evidence for the importance of the type 2 protein secretion system (T2SS) Lsp, which was identified as contributing to fitness in cold water (10). We detected mutations in *lspI* and *lspC* reducing fitness in our Tn-seq screen performed at 42°C, indicating that the importance of T2SS might not be limited to cold water. Our work further supports a previously described role for EnhB/C in maintaining envelope integrity (50) and adaptation to freshwater (20).

Previous predictions of genes important for water survival based on analyses differential transcription in response to water stress have been partially supported by the work reported here. For instance, there is a general trend of gene downregulation after introduction into water that is countered by upregulation of *enh* genes, which we have shown are essential for viability after water starvation (20). On the other hand, 20 of the candidate essential genes identified in this work were >4-fold downregulated after 24 h starvation in that study (20) (see Table S3). This indicates that although essential persistence factors may have low expression levels during starvation, they are doing so in a background of general transcriptional repression. In addition, there is the paradoxical result that six genes identified as essential for water stress resistance in our work, including *ppsA* (*lpg0805*) and *mip* (*lpg0791*), were upregulated in Δ*letS* cells after experiencing water stress for 2 h (14). This is surprising and suggests that the transcriptionally negative effect of *letS* on these genes in water may be transient (51), further arguing that the time point of transcriptional analysis after starvation is important for the analysis. This is in line with another study analyzing the transcriptome of RpoS mutants in water (25), in which three of our candidate genes, *ykuD* (*lpg1697*), *uvrB* (*lpg0072*), and Lon protease gene (*lpg1859*) are activated, while two, i.e., *ppsA* and *enhC*, are repressed by RpoS. Our study suggests that the absence of either of these genes results in an attenuated capability to persist, so there may be temporal regulation of these genes that has been previously unappreciated.

10.1128/msphere.00454-22.5 TABLE S3 Candidate genes transcriptional and proteomic data. Download Table S3, XLSX file, 0.02 MB. Copyright © 2023 Aurass et al. 2023 Aurass et al. https://creativecommons.org/licenses/by/4.0/ This content is distributed under the terms of the Creative Commons Attribution 4.0 International license.

One of the most striking results of this screen was the identification of numerous genes critical for respiratory chain function, mostly related to cytochrome *c*. This undoubtedly reflects the ATP-limiting conditions encountered during extended water exposure and highlights the critical roles that the maintenance of membrane potential, as well as intact ATP homeostasis, play to support survival in water starvation, as demonstrated in earlier studies (52, 53). Interestingly, depletion of intracellular ATP has been correlated with high drug persistence (54, 55). Concordantly, respiratory chain mutations were recently identified in screens for strains having high drug persistence, and a model linking defects in respiratory chain function to cytoplasmic acidification, biosynthetic imbalance, and eventual hyperstimulation of the RpoS response has been proposed (56). Strikingly, our screen for genes whose interruption is lethal in a growth-limiting condition uncovered multiple respiratory chain genes which highlights the costs associated with drug tolerance and exemplifies the selective forces favoring phenotypic variation over genetic mutation in persister cells (57).

Maintenance of cell envelope integrity is a key microbial strategy for stress adaptation (58–60). Therefore, a putative cell envelope-associated Lpg1697 that is essential for persistence in water attracted our attention. Members of this family are present in a wide range of bacteria and can act as l,d-transpeptidases (LDTs) catalyzing l-d type peptidogylcan cross-links that act as alternatives to d-d type cross-links made by penicillin-binding proteins. The catalytic YkuD domain proteins use an active site cysteine and usually have a two-domain architecture with an N-terminal LysM peptidoglycan-binding domain and a C-terminal catalytic domain. With the exception of carbapenems, most β-lactam antibiotics cannot access the active site, allowing activity in the presence of most β-lactams (61, 62). Therefore, loss of this protein is predicted to have little effect on bacterial sensitivity to carbapenems but should result in loss of a critical barrier to protecting against carbenicillin sensitivity. Consistent with Lpg1697 having an important physiological function, hypersensitivity to carbenicillin was observed after deletion of this protein (Fig. 5) (41, 63). Enhanced sensitivity as a consequence of the loss of LDT activity has been implicated in protection against envelope stress in other organisms (64–66). In Mycobacteria it was suggested that d-d linkages are likely less flexible than l-d linkages, so the interplay between these two cross-links could be an important determinant of the stress response to hypo-osmolar conditions (67). Indeed, we observed a severe persistence defect of both cr*lpg1697* and *Δlpg1697* strains, with rapid loss of culturable bacteria after prolonged water stress (Fig. 4 and 5).

Despite the clearly definable phenotypes with characteristics typical for loss of l,d-transpeptidase function, murein profiling showed no detectable compositional differences of *Δlpg1697* relative to the WT. Although this failure could be due to limitations of the technique, it should be noted that there are 10 *L. pneumophila* genes encoding putative YkuD domain proteins that could compensate for loss of *lpg1697* and mask alterations in peptidoglycan profile (see Fig. S2). However, given the clearly definable phenotypes resulting from loss of Lpg1697 activity, there could be an alternative function unrelated to LDT activity and not detectable by muropeptide profiling. As is true for other bacteria lacking Braun’s lipoprotein (Lpp), *Legionella* species employ outer membrane beta-barrel proteins for cell wall anchoring of the outer membrane in a process that partially depends on LDT activity (37). It is possible that Lpg1697 is involved in cross-links that are specific for anchoring activities constituting only a small fraction of the total murein cross-links in the envelope. The phenotypes observed in this work could originate from a poorly tethered OM resulting from a spatially restricted defect in cross-linking.

In conclusion, the strategy described here is the first exhaustive description of *L. pneumophila* proteins essential for persistence in water that are dispensable for growth in culture. This study pointed toward the importance of the electron transport change in maintaining viability, as well as the identification of a putative Ldt that likely acts to maintain cell envelope integrity. Surprisingly, this protein is also required for intracellular growth, providing a surprising link between surviving hypotonic stress and establishing an intracellular niche.

## MATERIALS AND METHODS

### Bacterial strains and growth conditions.

Throughout the study, derivatives of L. pneumophila Philadelphia-1 strain Lp02Thy^+^ were used (30). In Lp02Thy^+^, the chromosomal *thy* mutant allele in the parental Lp02 strain was replaced with a functional *thyA* allele by allelic exchange using pJB3395, as previously described (68, 69). L. pneumophila strains were cultured at 37°C in BYEα broth or BCYEα agar plates (70) containing 0.4 mg/mL l-cysteine sulfate (Sigma), 0.135 mg/mL ferric nitrate (Sigma), 0.1% (wt/vol) α-ketoglutarate, and, when appropriate, 0.1 mg/mL thymidine (Sigma), 40 μg/mL kanamycin, or 5% sucrose. Plasmids were introduced into L. pneumophila by electroporation (71).

Escherichia coli strain DH5α λpir was used for all cloning. E. coli strains were grown in Luria broth (LB) or on solid LB plates supplemented with 50 μg/mL ampicillin, 25 μg/mL chloramphenicol, or 50 μg/mL kanamycin when appropriate. Bacterial strains used in this study are summarized in Table S4. Primary BMDMs from A/J mice were isolated and cultured as previously described (30). A. castellanii (ATCC 30234) was cultured as previously described (72).

### Construction of transposon mutant libraries.

Mutagenesis of L. pneumophila Lp02Thy^+^ with the Mariner *himar1* transposon was performed by electroporating pTO100MmeI in triplicate, as described previously (73, 74). Transformants were selected on 150-mm agar plates containing kanamycin. After colonies were washed off with BYEα medium, individual pools were stored at −80°C after adding glycerol to 25% (vol/vol).

### Illumina library preparation and sequencing for Tn-seq.

Bacterial genomic DNA was extracted with a DNeasy kit (Qiagen) and quantified by a SYBR green microtiter plate assay. Tagmentation and amplification of transposon-adjacent DNA for Illumina sequencing was conducted using a modified Nextera DNA Library Prep method, as described previously (75). Samples were multiplexed, reconditioned, and size selected (250 bp or 275 to 600 bp; Pippin HT) before sequencing (single-end 50 bp) using custom primers (75, 76) on a HiSeq2500 with high-output V4 chemistry at the Tufts University Genomics Core Facility.

### Establishing water cultures.

For the Tn-seq screen, pools of transposon mutants were directly inoculated from frozen stocks, adjusted to an *A*_600_ of 0.2, and grown overnight to an *A*_600_ of 3.8. Subsequently, cultures were washed twice in sterile tap water and inoculated to an *A*_600_ of 0.03 in 200 mL of water in 250-mL flasks. All tap water used was first passed through a 0.2-μm-pore size filter (Steritop; Millipore) and then autoclaved (77, 78). Next, 250-mL flasks were autoclaved twice with tap water prior to usage. Water cultures were stored in a humidified incubator at 42°C. Before plating, flasks were carefully swirled 25 times.

For confirmation of candidate genes by CRISPRi, single colonies of L. pneumophila Philadelphia-1 Lp02*thyA*::*dcas9* P*ahpC*::*luxCDABE* containing a single CRISPRi construct were grown and processed as described above with the difference that the culture volume was reduced to 20 mL of water held in 50-mL glass tubes. For evaluation of the long-term efficiency of CRISPRi with the *dotO* gene, the water culture was established in an identical fashion with the difference that an ambient temperature (22°C) was used for incubation to allow for assessment of the phenotype in a time frame exceeding 50 days.

### Generation of *L. pneumophila* CRISPRi strains.

Plasmids encoding crRNAs for targeting single ORFs by CRISPRi were constructed as described elsewhere (29). Oligonucleotides containing BsaI overhangs were ordered from Integrated DNA Technologies (IDT), duplexed, and ligated into pMME1540BsaI to create preliminary plasmids, which were confirmed by Sanger sequencing. Final plasmids were constructed by introducing pMME985 (tracrRNA) and a preliminary plasmid into pMME977 carrying a *thyA* gene by using the Gateway LR reaction. After confirmation of single clones, the final plasmids were electroporated into L. pneumophila Philadelphia-1 Lp02dCas9Lux and selected on plates without thymidine (29) (see Tables S2 and S6).

10.1128/msphere.00454-22.8 TABLE S6 Plasmids and strains used. Download Table S6, XLSX file, 0.01 MB. Copyright © 2023 Aurass et al. 2023 Aurass et al. https://creativecommons.org/licenses/by/4.0/ This content is distributed under the terms of the Creative Commons Attribution 4.0 International license.

### Tn-seq data analysis.

Sequencing reads were processed using FASTX-toolkit and BWA (Burroughs-Wheeler Aligner), and the built-in aligner in TPP was used to map reads into the chromosome (AE017354). Preprocessed reads were further analyzed by TRANSIT using the GUMBEL subroutine (24). Transposon insertion sites located in the 5′- and 3′-terminal 10% of the ORF were excluded, and GUMBEL was used to determine conditional gene essentiality.

### Molecular cloning and mutant construction.

Gene deletions were conducted by tandem double recombination using the suicide plasmid pSR47s as described previously (79); all strains used are listed in Table S4. Plasmids were propagated in E. coli DH5α λpir (80). For complementation, the *lpg1697* gene was integrated into the intergenic region between *lpg2528* and *lpg2529*, as described previously (38). All plasmids were confirmed by sequencing and are listed in Table S4.

### qRT-PCR gene expression analysis.

Bacterial cultures were fixed in bacterial RNA Protect reagent (Qiagen) at the time point of water culture inoculation and stored at −20°C until use. RNA was extracted by using an RNeasy kit (Qiagen, 74106), and cDNA was synthesized by using SuperScript VILO cDNA kit (Invitrogen, 11754050). Subsequently, cDNA was amplified with PowerUp SYBR green Master Mix (Applied Biosystems, A25742) in a StepOnePlus real-time PCR system (ABI, 4376600) according to the manufacturer’s instructions. Primers were designed for amplification of 150- to 200-bp fragments using the Geneious software package and are listed in Table S2. Controls, including the absence of reverse transcription, were included to confirm the absence of gDNA contaminants. For normalization, *rrnB* (encoding 16S rRNA) primers were included. Transcript levels of each targeted gene were calculated by using the 2^–ΔΔ^*^CT^* method relative to the reference strain carrying a nontargeting control plasmid.

### Intracellular growth assays.

The growth of L. pneumophila within A. castellanii and A/J BMDMs was monitored as described previously (30, 33, 74). If cocultures were plated for CFU, cells were lysed by 0.1% saponin followed by pipetting.

### Sacculus isolation and peptidoglycan analysis.

Peptidoglycan analysis was performed as previously described (81). In short, L. pneumophila cells were centrifuged, resuspended in phosphate-buffered saline plus 5% sodium dodecyl sulfate (SDS), and boiled for 2 h while stirring. To remove SDS from the sacculi, samples were ultracentrifuged and washed with MilliQ water several times. After the washes, sacculi were resuspended in 100 mM Tris-HCl (pH 8) buffer with proteinase K (20 μg/mL) and incubated at 37°C for 1 h. After that, the reaction was stopped by adding SDS (1% final concentration) and boiling at 100°C for 5 min. SDS was removed using multiple described washes, and sacculi were resuspended in water. Muramidase was added to the samples, which were incubated at 37°C overnight, solubilizing muropeptides from the sacculi. Soluble muropeptides were reduced by adding NaBH_4_, and the pH was adjusted to 3. Muropeptides were separated using ultraperformance liquid chromatography (Waters) and later identified using a matrix-assisted laser desorption ionization-time of flight mass spectrometry system (Waters). Triplicates from the peptidoglycan profiles for each representative strain were chosen for quantification, and the area under each peak was integrated for statistical analysis.

### Osmotic tolerance studies.

Osmotic challenge assays were performed as described previously with modifications (82). Briefly, fresh postexponential phase cultures (optical density at 600 nm [OD_600_] 3.5) were adjusted to an OD_600_ of 0.3, and 1-mL suspensions were pelleted by centrifugation at low speed (5 min, 5,000 × *g*). Subsequently, supernatants were removed and replaced with the indicated stress solutions and distilled water as a control. Cells were incubated under these conditions for 30 min in a shaking incubator at 37°C. Viability was determined by spot plating dilution rows on BCYEα agar plates.

### Antimicrobial susceptibility testing.

Disc diffusion assays were performed as described previously with modifications (83). Briefly, 6-mm filter discs were impregnated with 5 μL of a freshly prepared imipenem solution (2 mg/mL in H_2_O) or 5 μL of a 50-mg/mL carbenicillin solution, respectively. The discs were then placed on fresh BCYEα agar plates containing a fresh layer of the indicated *Legionella* strain. After 3 days of incubation, the zones of inhibition were determined.

The carbenicillin MIC was determined by microtiter plate assays, as described previously with modifications (84). Briefly, two single colonies of each L. pneumophila strain were pregrown to the exponential growth phase in BYEα broth and back-diluted to an OD_600_ of 0.15. Then, 100-μL aliquots of this suspension were distributed into a 96-well plate preloaded with a concentration gradient of serial 2-fold carbenicillin dilutions in BYEα (100 μL/well). The plates were grown for 46 h in a plate reader with continuous shaking (Tecan Infinite 200 PRO), and bacterial growth was recorded as the OD. MICs were determined as the lowest antibiotic concentration for which no growth of L. pneumophila was detected after 46 h at 37°C. Wells containing BYEα broth or bacterial suspension without antibiotic were used as negative and positive growth controls, respectively.

### Reproducibility.

The data gathered from long-term H_2_O incubation of Tn-Seq pools were the result of one experiment performed using three independently generated pools that were incubated in parallel during the long-term experiment. These pools were subsequently analyzed as a single combined superpool during bioinformatic analysis. Confirmation of long-term efficiency of CRISPRi depletion of the *dotO* gene was performed once. Long-term survival analysis of CRISPRi-depleted strains in water was performed two times independently with similar results. All other presented data are from one experiment of two or three independent repetitions.

### Data availability.

The data for this study have been deposited in the NCBI sequence read archive under accession number PRJNA816157.
