# Supplementary figures and images for: Identification of Genes Required for Long-Term Survival of Legionella pneumophila in Water
Source: mSphere. 2023 Mar 29;8(2):e00454-22. doi: 10.1128/msphere.00454-22 (PMC10117105; doi:10.1128/msphere.00454-22)

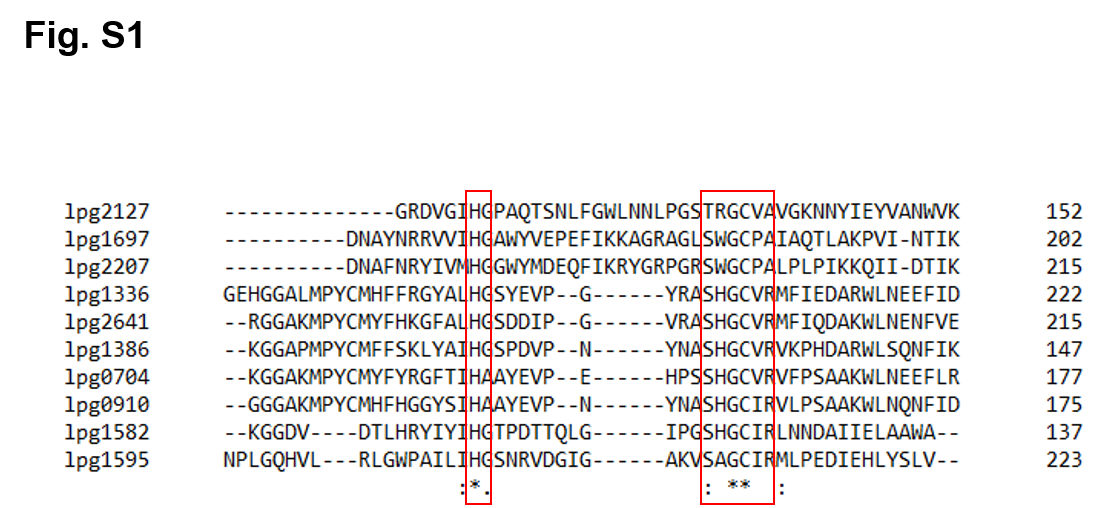

Supplement: FIG S1 [file msphere.00454-22-s0001.tif]

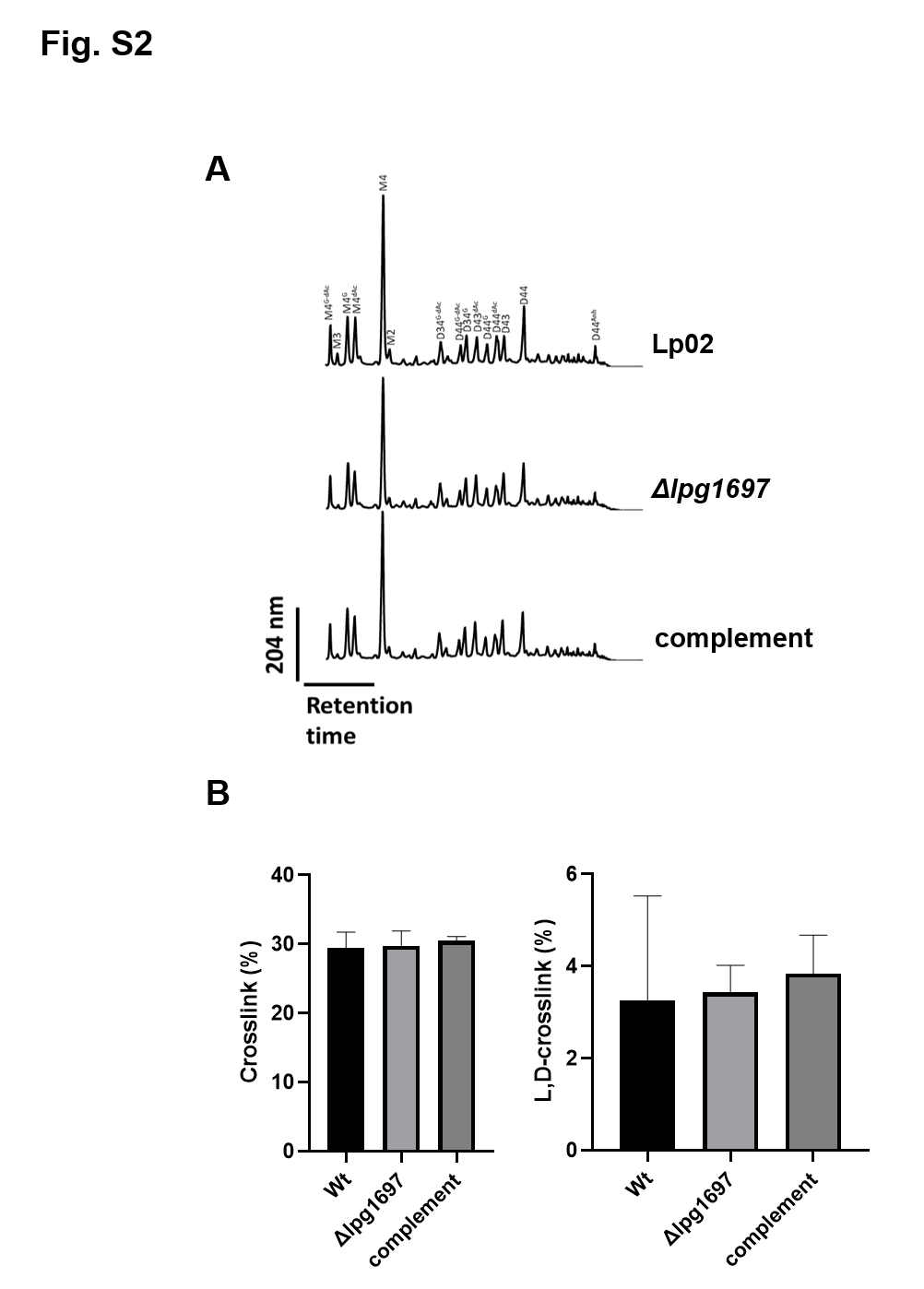

Supplement: FIG S2 [file msphere.00454-22-s0002.tif]
